# Acceptance of Fresh Pasta with β-Glucan Addition: Expected Versus Perceived Liking

**DOI:** 10.3390/foods9070869

**Published:** 2020-07-03

**Authors:** Danuta Jaworska, Maria Królak, Wiesław Przybylski, Marzena Jezewska-Zychowicz

**Affiliations:** 1 Department of Food Gastronomy and Food Hygiene, Institute of Human Nutrition Sciences, Warsaw University of Life Sciences-SGGW, Nowoursynowska 159c St., 02-776 Warsaw, Poland; wieslaw_przybylski@sggw.pl; 2 Department of Food Market and Consumer Research, Institute of Human Nutrition Sciences, Warsaw University of Life Sciences-SGGW, Nowoursynowska 159c, 02-776 Warsaw, Poland; maria.krolak@gmail.com (M.K.); marzena_jezewska_zychowicz@sggw.pl (M.J.-Z.)

**Keywords:** pasta, dietary fiber, β-glucan, consumers, perceived liking, expected liking

## Abstract

The aim was to recognize the effect of oat β-glucan fiber addition on expected and perceived liking of fresh pasta. Durum wheat semolina was enriched with oat β-glucan fiber in proportions: 0, 4, 8, 12 and 16% in relation to wheat flour. The evaluation of pasta samples was carried out by a group of 150 consumers and 10 panelists. Consumers evaluated pasta samples to assess the expected and perceived liking and the trained panelists established sensory profile.The consumers’ evaluation of cooked pasta with different oat β-glucan fiber additions showed that the sample with 16% oat β-glucan fiber addition was most liked (6.4 in a 1–9 scale). Consistency between expected and perceived liking increased with the increase in oat β-glucan fiber addition and was the highest for the sample enriched with 16% β-glucan. The sensory profile established by the trained panelists compared with the results of the consumers’ assessment showed that an increase in intensity of bran odor and flavor (up to value 4.08 for odor note and 5.31 for flavor) could have been related to higher perceived liking by the consumers. The increased amount of oat β-glucan fiber powder (16%), which provides fiber-rich products, is a promising ingredient for fresh pasta formulation.

## 1. Introduction

Typically, pasta is recognized as a unbalanced product due to the poor nutrition value of its proteins and low content of dietary fiber [1]. Due to the fact that a recent FAO/WHO (Food and Agriculture Organization of the United Nations and World Health Organization) report recommends an increase in the consumption of fruit, vegetables and wholemeal cereal products in order to increase the total dietary fiber intake to a minimum of 25 g per day [2], it would be beneficial to enrich pasta with this ingredient.

Pasta can be enriched with different sources of fiber, resulting in reduced calorie intake as an outcome of the manipulation of starch degradation [3,4]. The health benefits of dietary fiber are well documented [5]. Nonetheless, the addition of fiber can also have negative health and technological consequences. Fiber, as a component of the outer part of the grain, may contain some heavy metals or residual pesticides. It can also reduce the absorption of vitamins, minerals, and proteins [6]. Moreover, product modifications may adversely affect not only its physical and chemical properties, but also its sensory properties, which may be crucial as far as consumers’ acceptance is concerned [7,8]

Hence, improving pasta through the addition of dietary fiber has been pursued in recent years [1,9,10,11,12,13]. Oat bran can be used to provide foods with functional properties [14]. Espinosa-Solis et al. [12] proposed pasta enriched with oat bran as an alternative for people with special nutritional requirements (e.g., suffering from diabetes and overweight) due to the low content of starch and high content of dietary fiber. It seems that by combining the benefits of pasta (low glycemic index) together with the benefits of dietary fiber, healthy food products may be developed [15]. Focusing on the possibility of adding dietary fiber components to cereal products seems to be of interest both for consumers and the food industry [16,17].

Fiber enrichment in pasta is yet to be explored due to the still small amount of research on how fiber additions affect pasta quality, cooking characteristics, color, and sensory acceptance [18]. Moreover, the perception of fiber as an ingredient deteriorating the palatability of the product [19] requires searching methods and forms of fiber enrichment that do not give this negative effect. Previous studies have shown that pasta enriched with various forms of fiber was generally not preferred by consumers due to inferior texture, flavor and color [20]. High hardness and surface stickiness (due to extensive starch leaching) in pasta were indicated as undesirable properties [20,21]. These properties are generally associated with the disruption of the continuity of the gluten matrix during dough formation by insoluble fiber particles [11], which results in quality deterioration of the finished product [10,22,23].

The use of oat β-glucan fiber powder instead of oat bran in pasta production could be more justified, thanks to both nutritional and technological benefits [24] and because of physical and chemical properties [25]. Many health benefits of β-glucan are documented in the literature. Cereal’s β-glucan is highly effective in lowering both LDL (low-density lipoprotein), total cholesterol and serum triglycerides [26]. Consumption of dietary fiber including β-glucan may be helpful in reducing bowel transit time, preventing constipation, reducing risk of colorectal cancer and promoting the growth of beneficial gut micro flora, but also in leveling of postprandial glucose level and preventing coronary heart disease [27]. In addition to health properties that are important to consumers, β-glucan attracts food processors because of its stabilizing and thickening properties as well as its gelation and emulsification properties. Thus, β-glucan is a vital factor affecting enriched product quality [28].

The results of some studies demonstrated that fiber-enriched pasta can be produced when an additive is properly chosen and the amount added into the regular semolina-based pasta formulation is checked [18]. The results of the studies on the impact of oat β-glucan fiber powder addition on the cooking quality and physical properties of pasta are already available [25,29]. However, there are very few studies in which β-glucan was added to fresh pasta [30,31], especially studies that include a sensory evaluation of the product by a consumer group. However, fresh pasta could be more attractive to the consumer because the product is more tender and it takes approximately half of the time to cook it. Currently, consumers have little time to prepare meals; hence, they are proactively looking for convenient food [32]. On the other hand, growing interest in healthy eating is observed [33]. Fiber-rich fractions added to fresh noodles provide food convenience thanks to improved nutritional quality, shorter cooking time and acceptable cooking quality [31].

Sensory acceptance of a new product with β-glucan fiber addition is needed for market success. In the product development phase, a trained panel usually evaluates its properties [34]. Whenever only sensory descriptive analysis is conducted, researchers can understand the attributes and intensities of the sample and potentially differentiate among samples with different ingredients. However, researchers cannot determine which attributes are related to consumer acceptance, so for the deepest understanding of products, both methods should be used [35,36]. In the case of fiber-rich pasta, trained panelists assessed that the samples had a more bitter and branny flavor, more rough texture, significantly lower firmness and higher adhesiveness than the refined flour pasta [11,37]. At the same time, consumer studies on pasta with fiber show different results depending on the fiber source [38,39,40].

Visual impressions can affect expectations related to the quality of food products, in turn affecting the consumer’s attitude and leading to various purchasing choices and decisions (acceptance or rejection of food). All sensory properties of a product are experienced during its consumption. As research shows, expectations and sensory experiences are involved in the overall assessment of product quality [41]. At the same time, there is still little known about internal factors that affect food expectations [42].

Food characteristics, including flavor and appearance, together with brand name, price and health benefits, are important factors that influence consumer perception of products and their purchasing choices [43]. Additionally, the influence of eating habits on food choices and purchases is critical in regard to consumers’ acceptance of foods [44,45]. In consumer research, the sensory properties of newly developed functional products are usually evaluated and differences in assessment are sought for in relation to the sociodemographic characteristics of the group. However, the relationship between product acceptance/liking and experiences associated with previous consumption of this product and its conventional counterparts, as well as the consumers’ interest in a particular functional additive, is less known.

The relationship between consumers’ expectations towards food and experiences after consumption (i.e., satisfaction before and after purchase) is commonly believed to determine overall product satisfaction, and consequently, the probability of repurchasing the product [46]. It is known that the expected quality of a product increases with the perceived attractiveness of its appearance. The addition of fiber could have a positive impact on consumers’ expectations (i.e., bright yellow color [18]) but a negative impact on quality experience [8,45,47]. However, the sensory properties of the fiber-enriched pasta and its cooking quality depend strongly on the type of fiber source and its addition level [18,22]. On the other hand, some research [48] has indicated that there is no significant relationship between quality expectation and quality experience.

It is assumed that the product selected as the most liked one based on its appearance (high expected liking) would receive high scores after being tested (high perceived liking). In addition, it is expected that differences in fresh pasta sample selection based on appearance and in assessments after tasting depend on the frequency of pasta consumption and the interest in dietary fiber. It is hypothesized that consumers who frequently consume pasta and are highly interested in fiber content would be characterized by higher expected and perceived liking of pasta with a more substantial oat β-glucan fiber powder addition. Hence, the aim of this study was threefold: (1) to recognize the effect of β-glucan addition on the liking of fresh pasta in relation to consumers’ expectations and the perception of product after consumption; (2) to define the relationship between the appearance-based pasta selection process and the assessment of the product after consumption with the frequency of pasta consumption and dietary fiber interest; (3) to compare the sensory profile established by trained panelists with the results of consumers’ liking evaluation in order to understand which attributes could affect consumers’ liking.

## 2. Materials and Methods

### 2.1. Pasta Production

The pasta consisted of the wheat semolina, water and the oat β-glucan fiber powder. The wheat semolina was supplied by Assmannmühlen GmbH (Guntramsdorf, Austria). The composition of wheat semolina pasta was examined using near-infrared spectroscopy (NIR-Flex N-500, Buchi, Switzerland), and the following composition was observed: saccharides 71.4%, protein 12.3% (wet gluten 30.2%), water 13.1%, and ash 0.8%. The oat β-glucan fiber powder was supplied by a local producer (Microstructure Inc., Warsaw, Poland). The fiber preparation consisted of 44% dietary fiber (23% soluble and 21% insoluble fractions) and contained 16 g of β-glucan per 100 g of preparation.

Durum wheat semolina was mixed with oat β-glucan fiber powder in five proportions: without any fiber addition (P-0), with 4% addition (P-4), 8% (P-8), 12% (P-12) and 16% fiber addition (P-16). Therefore, the study samples consisted of five samples that were coded for this manuscript with the letter ‘P’ and the level of fiber addition (%) as follows: P-0; P-4; P-8; P-12; P-16. The semolina, the oat β-glucan fiber powder and water were mixed in the pasta extrusion machine (P3, La Monferrina, Italy). The water content was adjusted to achieve 33% moisture in the blend. After mixing, pasta was extruded at 50 rpm through fusilli forming die in technological rooms located at the Institute in the Department of Technique and Food Development.

### 2.2. Sample Preparation and Experiment Design

The pasta (fresh—never dried) was cooked for the optimal time (about 2 min) established previously. The shortest cooking time was 110 s for the sample P-16. In the case of the tested pasta samples, the cooking time differed by a maximum of 10 s (unpublished data). After cooking, the pasta was tempered in cold water and submitted for assessment. The pasta prepared in this way was evaluated by both the consumer and the trained panelists.

The samples were prepared by placing 30 g of pasta in odorless plastic boxes of 150 mL volume and closed with a lid. Each lid was previously coded according to the planned scheme. The studied set consisted of a white tray, five properly coded pasta samples—control sample (without fiber addition) and four samples with different levels of fiber addition—a fork, paper napkins and water. The samples were served in a random order.

The cooked pasta samples were evaluated within two hours of being stored in room temperature (24 ± 2 °C). The assessment was conducted in rooms with daylight. The evaluation was carried out in a laboratory intended for sensory food evaluation, equipped in booths. The room was air conditioned, free from off-odors and with limited noise. The evaluators were separated from each other to carry out the assessment in full concentration. The walls of the room as well as all tables were white. The ambient temperature was 22–23 °C. The assessment and condition mode were determined in accordance with Meilgaard [34].

### 2.3. Consumers’ Study

#### 2.3.1. Study Group

The liking of the pasta samples was evaluated by a group of 150 participants, who declared they ate pasta and who were not on any kind of dietary restriction. Characteristics of the population involved in this study are presented in Table 1. Most of the participants (90%) were in the age 18–24 years and represented mostly young adult consumers, residents of towns with more than 500,000 citizens, from the central part of Poland. All participants gave voluntary consent to participate in the study in the form of a written agreement. The study was conducted in accordance with the Helsinki Declaration [49].

#### 2.3.2. Study Procedure

The research was carried out at the Warsaw University of Life Sciences at the facilities of Institute of Human Nutrition Sciences (laboratories for food evaluation and food preparation). The consumers were invited to participate in the study through an advertisement on the website and posters displayed at the university.

There were two stages in the consumer study. In the first stage, the participants answered questions concerning pasta consumption and presented their opinions about pasta. The questionnaire was prepared in Polish and translated for the purpose of this manuscript. The interest in the fiber content in the diet was evaluated using the following question: “Are you interested in the amount of fiber in your diet?” The willingness to purchase selected types of pasta was measured using three questions: “Are you willing to buy pasta with fiber addition/fresh pasta/pasta with a short cooking time?” For assessing pasta consumption two questions were used: “Do you eat pasta regularly?” and “Do you eat different sorts of pasta?” The participants answered these questions on a 5-point scale: yes (1), rather yes (2), neither yes nor no (3), rather no (4), and no (5). The importance of visual appearance and of the addition of fiber when deciding which pasta to purchase was measured on a 9-point scale from 1—very important to 9—unimportant.

Then, a set of 5 samples of raw pasta (P-0, P-4, P-8, P-12, and P-16) was presented to the consumers. Based on the visual assessment, they were asked to choose one sample that met their liking expectations best.

After a short break, in the second stage, the participants tasted the cooked samples and evaluated them. The perceived liking of the cooked pasta was established by evaluation of overall liking of the set of 5 cooked pasta samples on a 9-point hedonic scale, where 1—extremely dislike and 9—extremely like [34,50]. Each participant received a set of samples separately coded with three-digit codes generated by the ANALSENS program (CogITos, Sopot, Poland). The samples were served at the same time in a random order. The assessment and the condition mode were determined in accordance with Meilgaard et al. [34] and Baryłko-Pikielna and Matuszewska [51].

### 2.4. Trained Panel Evaluation

#### 2.4.1. Sensory Panelists

The trained panel consisted of 10 members (8 women and 2 men, age 28–58), who were extensively formally tested before being selected, according to the ISO procedure [52]. The panelists had 4 to 15 years of theoretical and practical experience with sensory procedures and sensory evaluation of different food products with various methods (including profiling). The assessors’ ability to differentiate product samples by different concentrations of volatile and non-volatile stimuli was verified. They possessed the necessary skills to describe the flavor, taste and odor attributes of different samples. They underwent descriptive tests with the use of a series of food products, in which they described the sensory characteristics of the samples. The distinguishing marks were chosen, and their understanding was established and defined. Before proper evaluation, a set of pasta samples were presented to the panelists. Attributes were chosen by them, and a set of attributes was established and defined. During preliminary evaluation sessions, their proper understanding by the panelists was confirmed.

#### 2.4.2. Sensory Descriptive Analysis

The trained panelists established the sensory profile of the cooked pasta using the QDP (Quantitative Descriptive Profile) method [53].

Descriptors for the QDP method were chosen and defined during a panel discussion and then, verified in a preliminary session. Finally, 13 sensory attributes were measured to quantify the quality of the tested samples: 5 odor attributes—pasta, sweet, cereal bran, sour and off-odor; 1 attribute of color—intensity; 2 texture attributes—hardness and adhesiveness; 5 attributes of flavor—pasta, sweet, cereal bran, salty and off-flavor. On the basis of the all abovementioned attribute characteristics, the sensory trained panel indicated an overall sensory quality (low–very high) for each sample on a separate scale. Their definitions were agreed upon after being discussed by the panel members to ensure that the attributes concern various (recognizable) sensations and are equally understood by all the panelists. Additional preliminary evaluation sessions on pasta samples were performed in accordance with the profiling method procedure. An unstructured, linear scale of 100 mm that easily converted to numerical values (0–10 conventional units c.u.) was used. The marks of anchors of the tested attributes were prevalently as follows: none–very strong. There were two exceptions: for color—dark–bright and for the texture attribute—low–high. Two independent sessions (replications) were conducted. The average result in profiling method was based on 20 individual results.

The samples were coded separately for each assessment with three-digit codes generated by the ANALSENS program (CogITos, Sopot, Poland). They were served at the same time in a random order. Serving samples were random and balanced within days to prevent a carryover effect (i.e., the impact of a previous sample on a subsequent sample). Between the subsequent evaluations, the assessors received water to neutralize the taste. The assessment and the condition mode were determined in accordance with Meilgaard et al. [34] and Baryłko-Pikielna and Matuszewska [51].

### 2.5. Data Analysis

The consumer evaluations were analyzed with the use of a one-way analysis of variance (ANOVA) between groups, with the pasta samples as a variability factor. As a post hoc test, an LSD (Least Significant Difference) was conducted. The relationship between the consumers’ choices regarding raw pasta and the assessment of the samples after cooking was calculated using Spearman’s rank correlation. The impact of the samples, the session and the panelists on the studied sensory traits was analyzed using multifactor analysis (ANOVA). The impact of the sensory session (two sessions) was insignificant for most evaluated attributes, which positively verifies the experience of the panel and assessments conditions. The α value 0.05 was used. Principal Component Analysis (PCA) was performed for the results obtained in the QDP method. All of the statistical analyses except for PCA were performed using Excel 10.0 (Microsoft, Redmond, WA, USA) and Statgraphics plus 10.0 statistical package (Statistical Graphics Corp., Redmond, WA, USA). PCA was performed using the ANALSENS program (CogITos, Sopot, Poland). Additionally, multiple factor analysis (MFA) for attributes of sensory analysis was applied using Statistica 13 version software (TIBCO Software Inc., 2017).

## 3. Results

### 3.1. Characteristics of Consumer Group Concerning Pasta Consumption

The participants showed a relatively high interest in the fiber content in their diet. Approximately 70% of them chose the answer ‘yes’ or ‘rather yes’ in regard to this matter. About two-thirds of the participants (65%) declared willingness to purchase pasta with fiber addition (answers ‘yes’ or ‘rather yes’); only 10% of the participants were not interested in such a product. The willingness to buy fresh pasta was declared by more than half (54%) of the participants. Similarly, 58% of the respondents indicated interest in purchasing pasta with a very short cooking time. More respondents ate standard pasta (56.7%) than different types of pasta (44.0%) (Table 2).

### 3.2. Consumers’ Expected Liking

Among the five presented samples of raw pasta, P-16 (with 16% oat β-glucan fiber powder addition) sample was chosen most often by the participants (33.0%). The P-0 and P-4 samples were chosen by 19.3% of the participants (for each sample) and P-8 and P-12 were recognized as the most liked only by 14% of the group (for each sample).

The declared interest in fiber content in diet significantly influenced the choice of the most liked raw pasta (F_(4,14)_ = 2.44; *p* = 0.05). The ones who chose raw pasta P-16 were significantly more interested in fiber content in their diet than the participants who chose P-0 sample as the most liked one. Other variables concerning the consumption of pasta showed no relationship with the sample selection.

### 3.3. Consumers’ Perceived Liking

**Figure 1:**
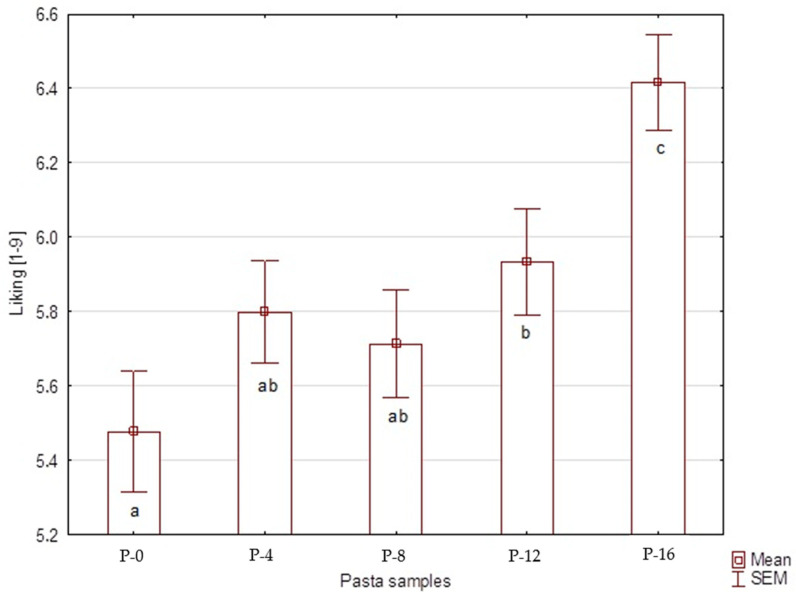
The consumers’ liking of the cooked pasta samples (*n* = 150); P-0—control sample, P-4, P-8, P-12, and P-16—4–16% oat β-glucan fiber powder addition; ^abc^ means with various letters, they differ significantly (*p* < 0.05), according to post hoc LSD (Least Significant Difference) test.

The mean scores of liking for the five pasta samples ranged between 5 and 7 on a 9-point scale (Figure 1). Differences in liking were found between samples (F_(4.74)_ = 5.67; *p* < 0.001). The pasta with 16% oat β-glucan fiber powder addition (P-16) was assessed more highly than the other samples. P-0 sample obtained the lowest hedonic score.

The participants who chose the sample of raw pasta as the one that meets their liking expectations best also assessed them better in liking evaluation after tasting (high scores—7–9). It was most common among the consumers who chose P-16 as the most liked sample of raw pasta (60.0%), and the least common among those who chose the P-8 sample (42.9%) (Table 3).

**Figure 2:**
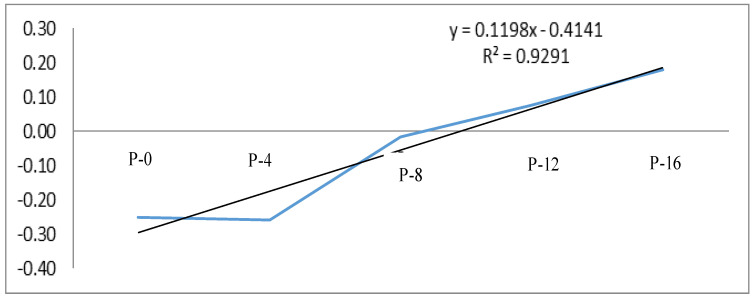
The relationship between consumers’ liking regarding raw pasta and the assessment of the samples after cooking; y axis represent a delta score; x axis present samples: P-0—control sample, P-4, P-8, P-12, and P-16—4–16% oat β-glucan fiber powder addition.

Figure 2 shows the relationship between the consumers’ liking of the raw pasta samples (based on the visual appearance) and the assessment of the cooked samples (the difference between expected and perceived liking). In the case of low oat β-glucan fiber powder content, there is some discrepancy, while in the samples with higher fiber content, there is consistency of expectations with the perception of product after cooking. The negative correlations regarding the samples P-0, P-4 and P-8, i.e., 0, 4 and 8% oat β-glucan fiber powder addition, showed that the consumers who chose raw pasta with low or no fiber addition, based on the appearance test, evaluated these samples less positively after they were cooked. The group indicating the raw pasta with a higher level of oat β-glucan fiber powder evaluated them highly after cooking.

**Figure 3:**
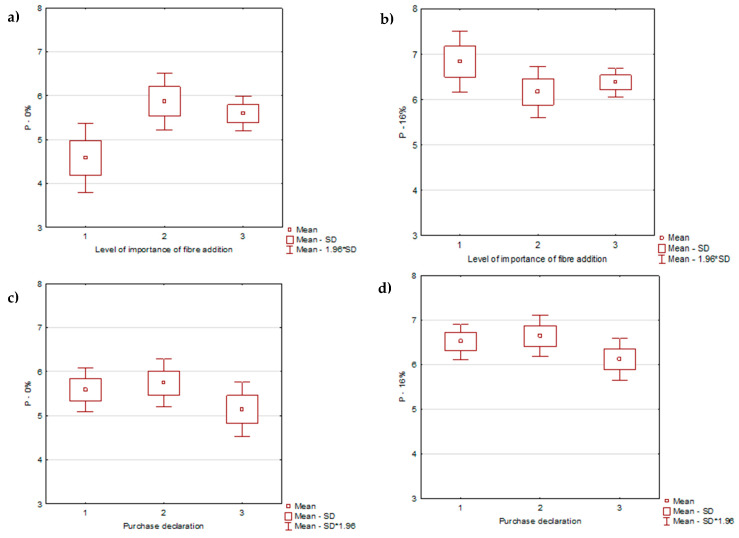
Results distribution of the liking evaluation of cooked pasta samples. (**a**): P-0 (control sample) and (**b**): P-16 (16% oat β-glucan fiber powder) in relation to the declared importance of fiber addition; (**c**): P-0 (control sample) and (**d**): P-16 (16% oat β-glucan fiber powder) in relation to the willingness to purchase pasta; (1) the addition of fiber in pasta was important, (2) the addition was moderately important, (3) the addition was not important.

Based on the consumers’ opinions, the participants were divided into three groups according to their opinions on the importance of fiber addition when buying pasta: (1) the addition of fiber in pasta is important (scores 1–3; 16.1% of the population); (2) the addition is moderately important (scores 4–6; 16.1%); (3) the addition is not important (scores 7–9; 67.8%). The distribution of perceived liking of the P-0 and P-16 samples is presented in Figure 3a,b, respectively. The group of consumers for whom fiber addition was important evaluated the P-0 sample lower than the other participants did (Figure 3a) and evaluated the P-16 sample slightly higher in relation to groups 2 and 3 (Figure 3b). Comparing the distribution of the liking of the samples P-0 (Figure 3c) and P-16 (Figure 3d), the perceived liking of both samples was assessed higher by the consumers who declared that fiber addition is of high or moderate importance.

### 3.4. Sensory Descriptive Analysis

Based on the assessment of 13 sensory attributes, significant differences were observed between the evaluated samples using the QDP method (Table 4). Most differences between samples occurred due to cereal bran odor, flavor and color intensity (*p* < 0.01). Moreover, significant differences in the intensity of pasta odor, flavor and off-flavor (*p* < 0.05) were observed within the studied material.

**Figure 4:**
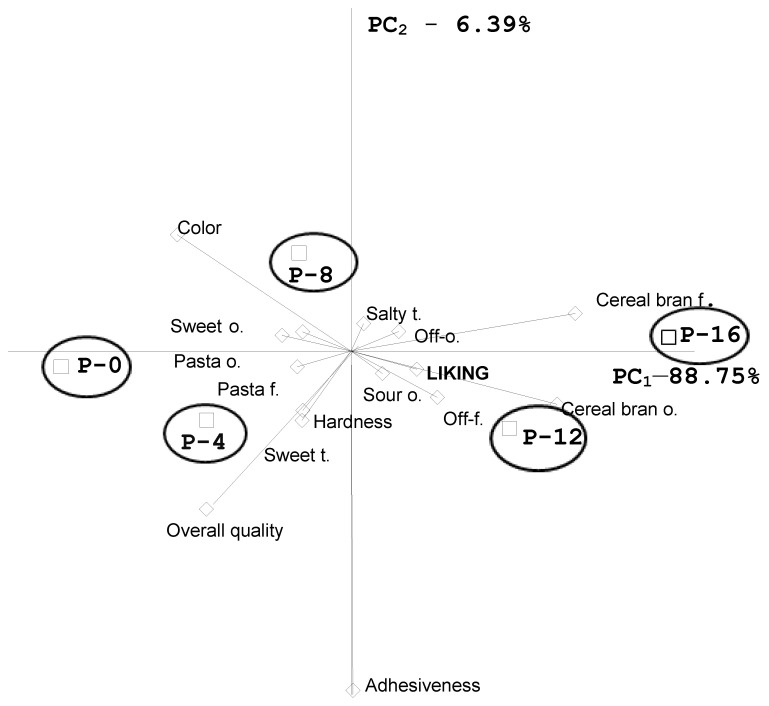
PCA (Principal Component Analysis) matrix of the mean sensory attributes obtained in the QDP (Quantitative Descriptive Profile) method for cooked pasta (P-0—control sample, P-4, P-8, P-12, and P-16—4–16% oat β-glucan fiber powder addition); o.–odor, f.—flavor, t.—taste.

Based on the results obtained using the QDP method, PCA was conducted and the obtained results are presented in Figure 4.

The first two principal components accounted for 95.14% of the total variability of all the sensory attributes, where the first component PC_1_ explains 88.75% of the variability. In this condition, the liking of pasta was strongly correlated with cereal bran odor of the studied samples. The cereal bran flavors and off-odor were the most important attributes characterizing samples P-12 and P-16. The vicinity of these two mentioned samples to the cereal bran odor and flavor vectors indicated the important relationship of these attributes as positive drivers of consumer liking. The position of P-8 samples indicated their different characteristic to samples P-0 and P-4 as well as to samples P-12 and P-16. Sweet and pasta flavors’ vector indicates that these attributes were positively correlated with each other and these attributes described the quality of samples P-0 and P-4 to the highest extent. We observed that the overall sensory quality obtained by the trained panel was associated positively with the sweet odor and taste of pasta, as well as texture attributes.

Similar results were also obtained for other multivariate analysis, i.e., multiple factor analysis (MFA) in the case of relationship of individual attributes of sensory quality with the main variables in the overall multidimensional variability (Table 5).

## 4. Discussion

In recent decades, the understanding of the importance of sufficient dietary fiber in the human diet has been increasing. The offer of whole grains on the market is growing; however, there is still a huge gap between recommended levels of dietary fiber intake and its consumption. Hence, improving various products, including pasta, through the addition of dietary fiber has been pursued in recent years [11,12,13,25,54].

In the current study, the participants’ choices based on the visual appearance (expected liking) of the raw pasta showed that the sample of pasta with the β-glucan fiber content of 16% was most desirable. Chillo et al. [55] emphasized that in the case of products of this type, the dark coloring is a positive trait, as consumers associate it with high-fiber products. However, Makhlouf et al. [18] have shown that a control pasta sample (without fiber addition) was preferred over any of the 15% fiber-enriched formulations. These findings can be explained by referring to the familiarity with the original color of the wheat or durum pasta, which was not conducive to acceptance at the very first introduction to the consumers. The opposite result of our study may be related with the addition of oat β-glucan, which influenced the color to a low degree. It was confirmed in the study regarding bread with oat β-glucan fiber powder addition [54,56]. Thus, the color change after adding fiber and an individual’s color preference have the potential to affect consumers’ liking [57]. Nevertheless, the possible cultural differences in the perception of the color of the pasta must be taken into account, and therefore, further research in different cultures is recommended. Such cultural differences were confirmed by Dean et al. [58] in the study on the perception of bread with fiber addition carried out in three European countries. The results presented in this study have shown that higher interest in dietary fiber correlated with the liking of cooked pasta with a 16% oat β-glucan fiber powder addition. Such choice could have been made due to many reasons, including the perception of fiber as a beneficial food ingredient [58]. Although information about the fiber content was not presented to the participants, the darker color of the samples with added fiber could suggest this addition [8,19]. Nevertheless, the assumption that the frequency of pasta consumption could be related to the sample selection has not been confirmed. No relationship has been found between these variables, which means that frequent pasta consumption is not a barrier for liking towards the modified pasta [18]. The presented results suggest that the addition of oat β-glucan fiber powder to pasta may contribute to its attractiveness to fiber-conscious consumers and not necessarily to those who often eat pasta. Research indicates that a growing group of consumers is interested in enriched food products of improved nutritional values [47]. Moreover, some studies indicated that proper labeling and marketing of the fortified products can positively influence acceptance of these products through building positive expectations [59].

The results presented in this study regarding the liking evaluation of the pasta samples showed that consumers gave higher scores of their perceived liking to the sample with a 16% oat β-glucan fiber powder addition. The study of Cecchi et al. [59] shows that fortifying pasta, bread, and granola bars with additives beneficial for health can only slightly affect their sensory profiles and slightly reduce their acceptance by consumers. Some negative drivers of liking, especially the darker appearance, earthy odor, flavor and the increase in bitterness do not cause product rejection by consumers. In previous studies on various grain products, fiber addition resulted in lower liking scores [7,19,47,60]. Deterioration of the sensory characteristics of the reformulated product as a result of the addition of substances with health-promoting properties [61,62] is noticed by consumers and may result in a decrease in the willingness to buy them. In previous studies [44,63,64], it has been shown that consumers are not willing to compromise the taste of functional foods even in exchange for health benefits. However, Verbeke [65] argued opposite, namely that consumers are able to accept the deterioration of the taste of functional foods to receive health benefits. Such an observation resulted from the present study. Despite these divergent results, it seems that when choosing functional foods, expectations and taste experiences are critical factors [44,64,65].

In this study, high scores of the sample with 16% oat β-glucan added were preceded by the choice of this sample as the most liked (expected liking) by the largest number of young consumers. It can be assumed that the appearance of the sample before cooking, primarily conditioned by its color, could also strongly affect the assessment after tasting. Perceived liking could, therefore, be the result of both taste and visual sensations. In addition, differences in perceived liking found in groups with different declarations regarding the importance of fiber addition in their purchase decisions may confirm the importance of consumer awareness in sensory evaluation [19,66,67,68], which can be a promising trend among young consumers.

The results showed that each group predicted the sensory attributes of the chosen product, i.e., high consistency between expected and perceived liking. Consistency increased with increasing oat β-glucan content and was the highest for the P-16 sample. In addition, young adult consumers who chose pasta with a lower oat β-glucan content were most often disappointed (higher difference between scores), while those who chose pasta with a higher oat β-glucan content reinforced their positive evaluation (small difference between scores). It must be noted that the expectations and perception in relation to the flavors of the product are critical factors when choosing a functional food [44], which in the case of the high liking of the P-16 sample, gives it a good chance of success on the food market. Proper marketing of the enriched products could set favorable expectations of those, thus, also improving their acceptance.

The liking of both samples with 12 and 16% oat β-glucan fiber powder addition was assessed higher by the young adult consumers who declared that the fiber addition is of high or moderate importance. Similar results were obtained in the study by Padalino et al. [69]. Consumers’ health consciousness influences their perceptions and purchase behaviors [70].

The trained panel evaluated the P-12 and P-16 samples, as those with higher intensity of cereal bran odor and flavor. According to the results obtained in the consumer group that was involved in the present study, assessment of higher oat β-glucan fiber powder addition improved pasta liking significantly for the sample P-16. Even lower hardness of these samples did not lower the level of liking. The attributes like pasta odor and flavor as well as sweet odor and flavor described the quality of the samples without and with a low fiber addition, i.e., P-0 and P-4.

It is important to point out that both assessments are still important to understand consumer expectation and perception of food products. The discrepancy between consumer results and results from a trained panel could be explained by the fact that young adult consumers might be ‘tired’ of traditional products and open or ready to purchase new or healthier products. A similar trend was observed with bread rolls enriched with 12% fiber [54] or bread enriched with 16% fiber [56] compared to a plain wheat product. The fiber-enriched rolls received higher scores of overall acceptance and more participants expressed their willingness to purchase such a product [54]. Moreover, Cecchi et al. [59] indicated in their study that approximately 30% of consumers preferred the fortified sample over the control one, including pasta, and 50% were willing to pay more for the fortified products. Furthermore, Bagdi et al. [47] in their study show that general consumer acceptance of modified pasta decreased with regards to more intense darker color, and more bitter and more sour taste. Acceptance is significantly higher among assessors, who usually buy unconventional pasta.

The positive aspect of this study was the evaluation of pasta with different oat β-glucan fiber powder addition simultaneously in a group of consumers and panelists in one study. Although the usefulness of these results obtained in consumer and trained panelist groups in the product development process is varied, the knowledge of their associations is valuable for better planning of this process, especially in a situation where consumers who took part in the study will remain for a long time as decision-makers on the food market. Moreover, consumer liking of pasta enriched with 12 and 16% oat β-glucan fiber powder addition indicates a possibility of nutritional added value product creation, which may in turn, constitute an additional fiber source in the diet. In the current study, we indicate that frequent pasta consumption is not a barrier for liking the modified pasta, which may encourage producers to introduce new healthy products. Despite this contribution to existing knowledge, the study has some limitations. One of them concerns the sociodemographic characteristics of the consumers’ group. This characteristic was not taken into account during the selection of the sample. As a result, the sample is not balanced in gender or age, which did not allow a reliable comparison of the results between groups. Moreover, only a few features of consumers were included in the analysis. There is a need to include more cognitive and emotional factors that may also modify expected and experienced liking of pasta with varied addition of oat β-glucan fiber powder and the associations between them. Although our findings have the abovementioned limitations, we hope the study provides an interesting insight into the liking of pasta with fiber addition and its determinants. In further research, it is recommended to compare results obtained in populations with different cultural backgrounds and corresponding sociodemographic characteristics.

## 5. Conclusions

The consumers’ evaluation of raw pasta with different oat β-glucan fiber powder addition showed that the sample with 16% oat β-glucan addition was most liked. The sample of the cooked pasta with the same addition was also assessed higher in liking score than the other samples tested by the young adults participating in the study. Thus, the study showed high consistency between expected (prior tasting) and perceived (post tasting) liking by the consumers. The consumers highly interested in fiber were characterized by higher expected and perceived liking of fresh pasta with a higher oat β-glucan addition, while such a relationship was not confirmed for those who frequently eat pasta. Thus, the study results only partially confirmed our hypothesis and showed that addition of oat β-glucan to pasta makes it more attractive to fiber-conscious consumers. Moreover, the sensory profile established by the trained panelists compared with the results from the consumers’ assessment showed that increase in intensity of bran odor and flavor could explain higher perceived liking by the consumers. Consistency between expected and perceived liking increased with the increase in oat β-glucan fiber powder addition and was the highest for the samples with 16% β-glucan. The substantial addition of oat β-glucan fiber powder (16%) is a promising ingredient for fresh pasta formulation, providing a fiber-rich product.

## Figures and Tables

**Table 1:** Characteristics of the study group (%).

	Total (*N* = 150)
**Gender** (%)	
Female	76.0
Male	24.0
**Age** (mean; standard deviation)	22.7; 3.5
**Education** (%)	
Secondary	78.7
Academic	21.3
**Place of residence** (%)	
Village	20.0
Town with less than 500,000 citizens	25.3
Town with more than 500,000 citizens	54.7

*N*—the number of participants.

**Table 2:** Characteristics of the consumer group regarding frequency of eating and willingness to purchase pasta, and interest in fiber.

	Participants’ Opinions from the 5-Points Scale (%)					Mean (SD)
	Yes (1)	Rather Yes (2)	Neither Yes Nor No (3)	Rather No (4)	No (5)	
Interest in fiber content in their diet	32.7	37.3	21.3	7.3	1.4	2.1 (0.98)
Willingness to purchase pasta with fiber addition	40.0	26.0	26.0	3.3	4.7	2.1 (1.10)
Willingness to purchase fresh pasta	30.7	26.0	28.0	9.3	6.0	2.3 (1.18)
Willingness to purchase pasta with a very short cooking time	30.0	27.3	27.3	12.0	3.4	2.3 (1.12)
I eat pasta regularly	30.0	26.7	23.3	12.7	7.3	2.4 (1.24)
I eat different type of pasta	24.0	40.0	10.0	22.7	3.3	2.4 (1.18)

SD—standard deviation.

**Table 3:** Distribution of liking scores of the cooked pasta sample with regards to the liking of the raw pasta sample.

	Range of Hedonic Scale *	Liking of the Raw Pasta Sample				
		P-0 % (*N* = 29)	P-4 % (*N* = 29)	P-8 % (*N* = 21)	P-12 % (*N* = 21)	P-16 % (*N* = 50)
Rates of the cooked pasta sample	1–3	10.3 (3)	6.9 (2)	19.0 (4)	4.8 (1)	6.0 (3)
	4–6	37.9 (11)	37.9 (16)	38.1 (9)	38.1 (8)	34.0 (17)
	7–9	51.7 (15)	52.2 (16)	42.9 (9)	57.1 (12)	60.0 (30)

* 9-point hedonic scale (1—extremely dislike, to 9—extremely like); 1–3 low scores, 4–6 average scores, 7–9 high scores; P-0—control sample, P-4, P-8, P-12, and P-16—4–16% oat β-glucan fiber powder addition; (*N*)—the number of participants.

**Table 4:** Results of variance analysis of data obtained in QDP method (Quantitative Descriptive Profile) for cooked pasta (*n* = 20).

Attribute Intensity (0–10 c.u.)	Pasta Samples *						
	P-0	P-4	P-8	P-12	P-16	F	p
Pasta o.	8.41 ^a^	7.54 ^ab^	7.47 ^b^	7.30 ^b^	7.08 ^b^	2.64	0.05
Sweet o.	3.04	2.99	2.81	2.30	2.39	1.06	0.37
Sour o.	1.26	1.22	1.20	1.49	1.71	0.55	0.69
Cereal bran o.	1.74 ^a^	2.13 ^a^	2.50 ^a^	3.86 ^b^	4.88 ^c^	15.64	0.01
Off-o.	0.75	0.67	0.85	1.03	1.46	1.06	0.38
Intensity of color	6.68 ^a^	5.57 ^ab^	5.75 ^ab^	4.69 ^b^	3.71 ^c^	7.68	0.01
Hardness	7.94	8.04	7.57	7.44	7.24	0.88	0.48
Adhesiveness	5.81	5.52	5.11	6.50	6.78	2.16	0.08
Pasta f.	7.92 ^a^	7.33 ^ab^	6.98 ^b^	6.70 ^b^	6.86 ^b^	1.57	0.05
Sweet t.	3.12	3.40	2.82	2.60	2.52	1.07	0.37
Cereal bran f.	1.75 ^a^	2.30 ^ab^	3.07 ^bc^	4.04 ^c^	5.30 ^d^	13.51	0.01
Salty t.	1.07	1.05	1.14	1.05	1.30	0.35	0.85
Off-f.	0.95 ^a^	1.15 ^a^	0.88 ^a^	1.20 ^a^	2.45 ^b^	4.91	0.01
Overall quality	7.72 ^a^	7.23 ^a^	6.23 ^b^	6.28 ^b^	5.25 ^c^	8.12 ^a^	0.01

* P-0—control sample, P-4, P-8, P-12, and P-16—4–16% oat β-glucan fiber powder addition; o.—odor, f.—flavor, t.—taste; ^a,b,c^ means with various letters, they differ significantly (ANOVA, *p* <0.05); c.u.—conventional units.

**Table 5:** The factor loadings of individual main components separated from MFA (multiple factor analysis) for attributes obtained in QDP method (Quantitative Descriptive Profile).

Traits	Principal Components			
	1	2	3	4
Pasta o.*	−0.84	0.23	0.20	0.45
Sweet o.	−0.90	0.27	0.24	−0.23
Sour o.	0.90	−0.10	0.30	0.29
Cereal bran o.	0.99	−0.12	0.05	0.09
Off-o.	0.96	0.15	0.19	0.16
Color	−0.96	0.22	−0.10	0.14
Hardness	−0.94	−0.17	0.24	−0.16
Adhesiveness	−0.04	−0.96	0.27	0.07
Pasta f.	−0.82	0.22	0.50	0.14
Sweet f.	−0.87	−0.16	0.32	−0.33
Cereal bran f.	1.00	−0.03	−0.02	0.00
Salty t.	0.76	0.56	0.29	−0.15
Off-f.	0.84	0.05	0.53	−0.04
Overall quality	−0.96	−0.17	0.14	0.17
Liking	0.95	−0.05	0.22	−0.22
Eigen value	11.60	1.59	1.15	0.66
% of variance	77.33	10.60	7.68	4.38

* o.—odor, f.—flavor, t.—taste
